# Tensile Creep Model of Slab Concrete Based on Microprestress-Solidification Theory

**DOI:** 10.3390/ma13143157

**Published:** 2020-07-15

**Authors:** Zhifang Zhao, Huanmi Zhang, Bo Fang, Yukun Sun, Yangfeng Zhong, Tao Shi

**Affiliations:** 1 College of Civil Engineering and Architecture, Zhejiang University of Technology, Hangzhou 310023, China; 2111706056@zjut.edu.cn (H.Z.); sunyukun@zjut.edu.cn (Y.S.); 2111706035@zjut.edu.cn (Y.Z.); 2 Wuhan Sanyuan Special Building Materials Co., Ltd., Wuhan 430083, China; fangbo@sanyuantc.com

**Keywords:** slab concrete, temperature–stress test, early age, tensile creep, hydration process, Kelvin model, temperature variation, microprestress-solidification theory

## Abstract

Tensile creep is an important factor affecting the early cracking resistance of concrete. The tensile creep model can effectively predict the development of tensile creep. In order to establish an appropriate tensile creep model, a temperature–stress testing machine (TSTM) was employed to test the development of temperature, deformation and restraint stress of benchmark concrete and concrete mixed with the MgO under different temperature curing modes. The development law of early age stress, strain and creep was analyzed via the test data of the TSTM. The early age tensile creep of concrete was predicted with the existing Kelvin creep model. The effect of variable temperature on creep was considered in this study, and an improved Kelvin creep model was proposed. The prediction accuracy of the two models was compared and analyzed. The results indicate that MgO has little influence on the creep and specific creep of concrete. The early age cracking resistance of MgO concrete is better than benchmark concrete. The improved Kelvin model based on the microprestress-solidification (MPS) theory predicts the early tensile creep of concrete more accurately in variable temperature conditions. These are significantly helpful for the application of the MgO expansion agent in dam engineering.

## 1. Introduction

The easy cracking property of concrete at an early age is always an issue in engineering. One of the effective methods to control the concrete cracking at early ages is to add an expansion agent [1,2]. Using an expansion agent can compensate for the early autogenous shrinkage of concrete and delay the occurrence of tensile stress, thus improving the cracking resistance of concrete. At present, traditional expansion agents generally include sulfoaluminate, aluminate clinker or CaO-based expansive additives, which are widely used in Japan, the USA and China [1]. In 1980, Mehta [3] proposed that the expansion of MgO can be used to compensate for the shrinkage of concrete, which provided a new idea for the preparation of shrinkage-compensated concrete. Mo et al. [1] found that, compared with traditional expansion agents, MgO expansion agents have advantages, e.g., they have a relatively lower water requirement, are chemically stable hydration products, namely Mg(OH)_2_, have a controllable expansion process and their delayed micro-expansion effect matches well with the concrete cooling phase. By adjusting the calcination temperature and holding time, the delayed expansion effect can be matched with the temperature shrinkage history of mass concrete, which can be used to compensate for the shrinkage deformation of mass concrete [4]. At present, the expansion principles of MgO expansion agents mainly include (i) crystal growth pressure caused by Mg(OH)_2_ growth and (ii) expansion pressure caused by very small Mg(OH)_2_ crystal water absorption. Chatterji [5] indicated that, during the hydration of MgO, a supersaturated solution of Mg^2+^ and OH^−^ would first form and then an Mg(OH)_2_ crystal would precipitate and grow in a confined region, resulting in crystal growth pressure, which leads to the expansion of the cement pastes. After more than 30 years of theoretical and practice research on MgO expansion agents in China, MgO expansion agents have been applied to more than 50 dam projects.

Creep is an inherent property of concrete. The creep of restrained concrete members can relax the most tensile stress, which reduces the concrete’s early cracking risk [6,7,8,9,10,11]. Therefore, it is essential to consider creep in the evaluation of concrete cracking resistance [12]. Research on concrete creep has been carried out continuously for decades [7]. Hatt first discovered the creep of concrete in 1907. To date, many scholars have been devoted to this field of research. With respect to the complexity of creep, current progress is insufficient, and concrete creep is not yet fully understood. Moreover, there were a few research studies on the creep of concrete mixed with MgO expansion agents. In order to accurately evaluate the cracking resistance of the early age concrete mixed with MgO, it is necessary to study its creep. This is of great significance for the application of concrete mixed with MgO in actual dam engineering.

Creep has a great influence on the deformation and mechanical properties of concrete. Based on creep testing, many scholars suggest various creep prediction models and improved models at early stages. At present, the most widely used tensile creep model is the B3 model [13,14,15]. The model dictates that the degree of hydration reaction of concrete is an important reason for its creep development. The prediction accuracy and application range of the B3 models are excellent, while the disadvantage of the B3 models is that it is difficult to apply it in actual engineering, due to its complicated calculation. De Schutter [16] proposed a Kelvin tensile creep model with variable parameters based on the degree of hydration. Wei et al. [17] improved the Kelvin tensile creep model by using three Kelvin units and established corresponding numerical calculation methods. Recent studies show that a single Kelvin unit in the tensile creep model based on the degree of hydration also predicts the development of the early concrete tensile creep accurately [17,18], and the use of multiple Kelvin units in the tensile creep model simulates the early tensile creep development further.

The benchmark concrete was called BC concrete for short. The MgO expansive agent was added to the benchmark concrete to obtain the expansion-agent concrete (EC). Under the temperature matched curing (TMC) mode and the constant temperature curing (CTC) mode, a temperature–stress testing machine (TSTM) was used to test the early stress and strain development of the two concretes in the restraint state. Based on the TSTM method of evaluating creep strain suggested by Kovler, the rules of tensile creep and specific creep were calculated and analyzed [19]. According to the test data obtained by the temperature–stress test (TST), the tensile creep model of the 3 Kelvin units was established based on the degree of hydration. For the same material, the model accuracy was tested via the TST, under different modes. The temperature changed during the TST, which affected the creep. In this paper, we take the variable temperature factors into consideration to improve the Kelvin model [17], and the accuracy of the model is verified. Finally, the prediction accuracy of the two models is compared and analyzed.

## 2. Materials and Methods

### 2.1. Materials and Mix Proportions

The BC concrete refers to the construction mix proportion of the slab concrete of rockfill dam. The EC concrete is the benchmark concrete mixed with the expansion agent. The mix proportions are shown in Table 1.

Raw materials: The cement used in this study was P.O 42.5 ordinary Portland cement. The fly ash was Class F I fly ash. The chemical composition of cementitious materials is listed in Table 2.

The fine and coarse aggregates were artificial gravel. Fine aggregate with a fineness modulus of 2.97 was employed. The apparent density, absorption and voids of sand was 2630 kg/m^3^, 1.70% and 44%, respectively. The mixture design used two coarse aggregate gradation. The small gravel with a 5–20 mm particle size accounted for 45% of the total coarse aggregate by mass. The medium gravel with a 20–40 mm particle size accounted for the other 55%. The apparent density and absorption of small gravel was 2620 kg/m^3^ and 0.61%, respectively. For medium gravel, the corresponding values were 2620 kg/m^3^ and 0.88%, respectively. The voids of mixed coarse aggregate totaled 38%.

Polycarboxylate high-performance water-reducing agent and air-entraining agent produced by Changan Yucai were used in this study. A certain M-type MgO expansion agent was used in the test. The performance indicators of the M-type MgO expansion agent are shown in Table 3. In addition, the restrained expansion rate of the MgO expansion agent at 7 days should not be less than 0.015% (0.030%) under 20 °C (40 °C); the difference of restrained expansive rate at 28 and 7 days should not less than 0.015% (0.030%) under 20 °C (40 °C).

### 2.2. TSTM Test

**Figure 1:**
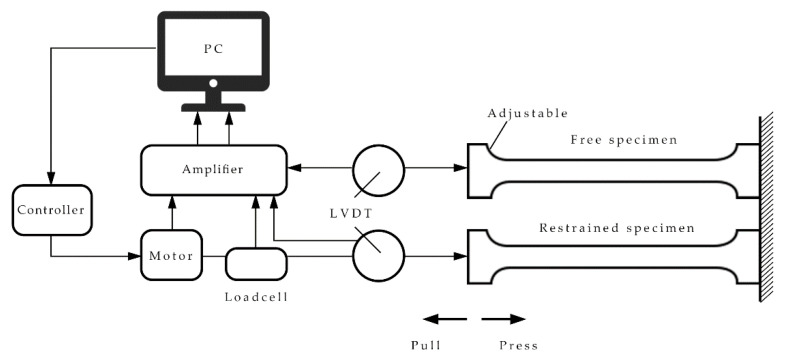
Temperature–stress testing machine (TSTM).

TSTM (shown in Figure 1) was employed in this study, as it is the testing principle and method proposed in the literature [20,21].

The temperature–stress test used two dog-bone-shaped specimens: one was a restrained specimen, and the other was a free specimen. The free and restrained specimens maintained the same temperature-curing histories, and both were enfolded with a plastic film, to prevent moisture exchange with the outside. Hence, the drying shrinkage of the concrete could be ignored [22]. The TMC mode and the CTC mode were adopted in this study. The temperature curve of the TMC mode was determined according to the highest temperature of the local construction site and the spontaneous heating capacity. In the constant temperature mode, the temperature of the specimens was kept at 20 °C. The temperature of the two modes dropped at the same equivalent age [23].

## 3. Results and Discussion

### 3.1. Properties of Fresh Concrete Mixes Studied

**Figure 2:**
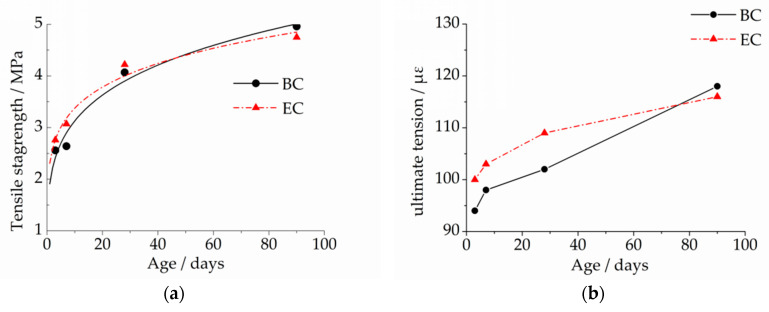

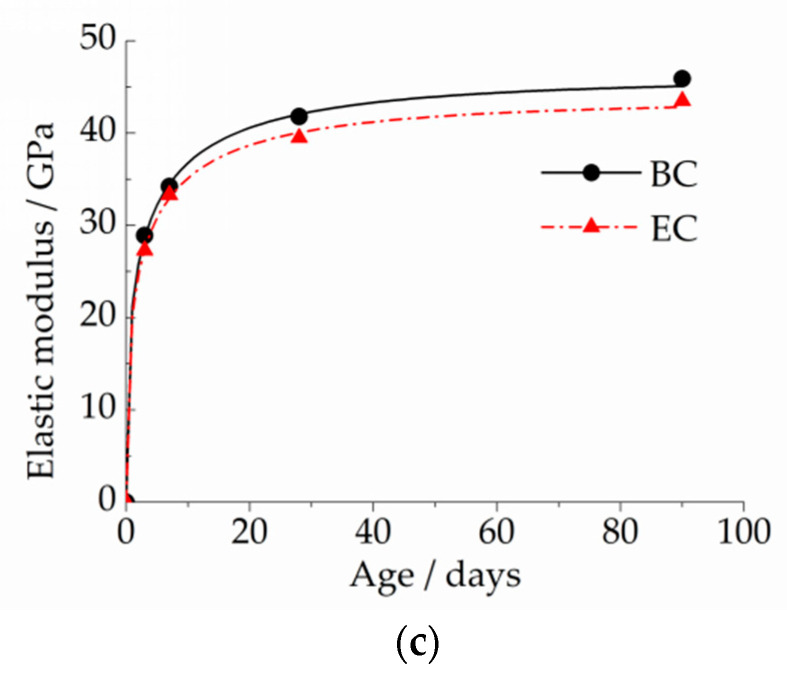
Characteristics of hardened concrete: (**a**) tensile strength, (**b**) ultimate tension and (**c**) elastic modulus.

Table 4 shows the properties of fresh concrete, and Figure 2 shows the mechanical properties of hardened concrete, such as tensile strength, ultimate tension and elastic modulus. It can be seen from Table 4 and the Figure 2 that the setting time of the two concretes was relatively close, and the slump and air content both met the performance index. The tensile strength of EC was greater than BC at 3, 7 and 28 days, and less than BC at 90 days. The development laws of ultimate tension and tensile strength of the two concretes were almost the same. The elastic modulus of EC was less than BC at 3 days, 7 days, 28 days and 90 days, and the elastic modulus of the two concretes was relatively close at an early age.

### 3.2. Development Curves under Different Temperature Curing Modes

**Figure 3:**
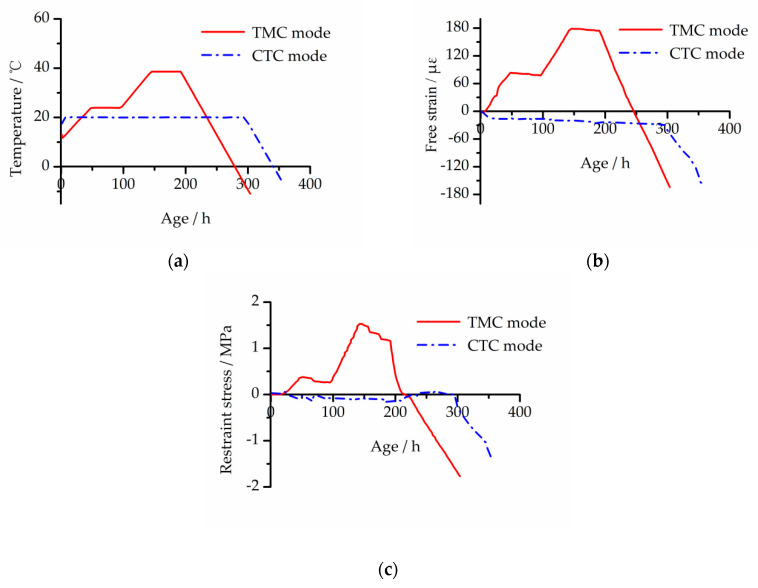
Development curves of BC under two modes: (**a**) temperature development, (**b**) free strain development and (**c**) restraint stress development.

**Figure 4:**
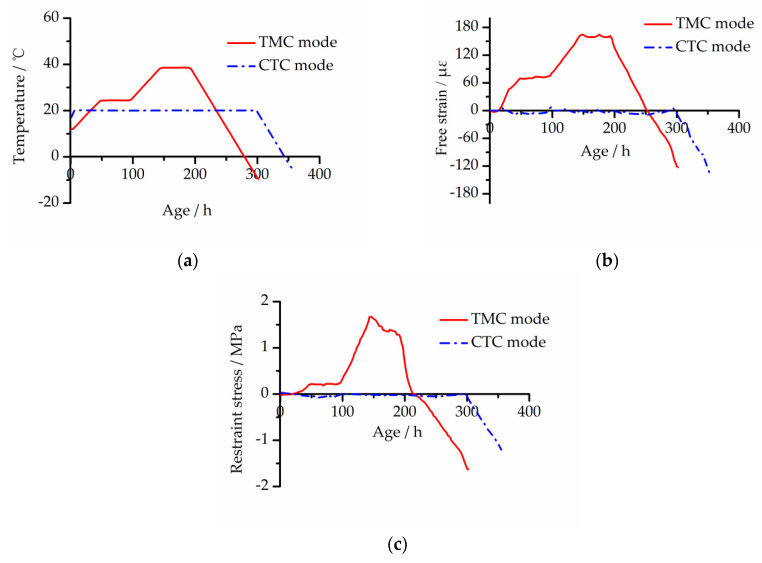
Development curves of EC under two modes: (**a**) temperature development, (**b**) free strain development and (**c**) restraint stress development.

During the TST, the computer recorded the temperature histories, free deformation strain (free strain) of the free specimen and restraint stress of the restrained specimen [20]. Figure 3 and Figure 4 show the development curves of temperature, free strain and restraint stress of the two concretes under two modes.

As shown in Figure 3b and Figure 4b, the final free strain of EC was smaller than that of BC under the two temperature curing modes, and the shrinkage reduction of TMC mode and CTC mode was 25.6% and 12.0%, respectively. Through analysis of the development procession of free strain under CTC mode, it is clear that the early autogenous shrinkage of the concrete with the added MgO expansion agent was compensated for, and the appearance of the tensile stress was delayed.

In the cooling phase, because concrete specimens under two modes were not broken at the lowest temperature of the TSTM, both were manually pulled off by the motor. The cracking stress of the TMC mode and CTC mode of the BC was −2.00 and −1.71 MPa, respectively; the cracking stress of the EC was −2.29 and −1.99 MPa, respectively. Combined with Figure 2a, the tensile strength of EC at an early age was significantly greater than that of BC. This shows that, whether using the TMC mode or CTC mode, the cracking resistance of concrete is significantly improved with the MgO expansion agent added.

### 3.3. Tensile Creep under Different Temperature Curing Modes

**Figure 5:**
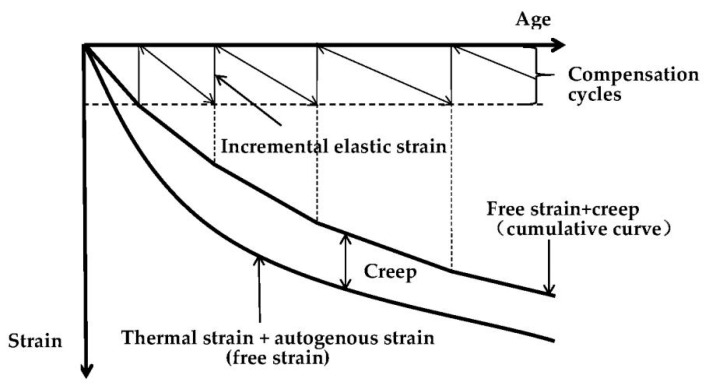
Calculation schematic diagram of tensile creep based on TSTM adapted from [19].

**Figure 6:**
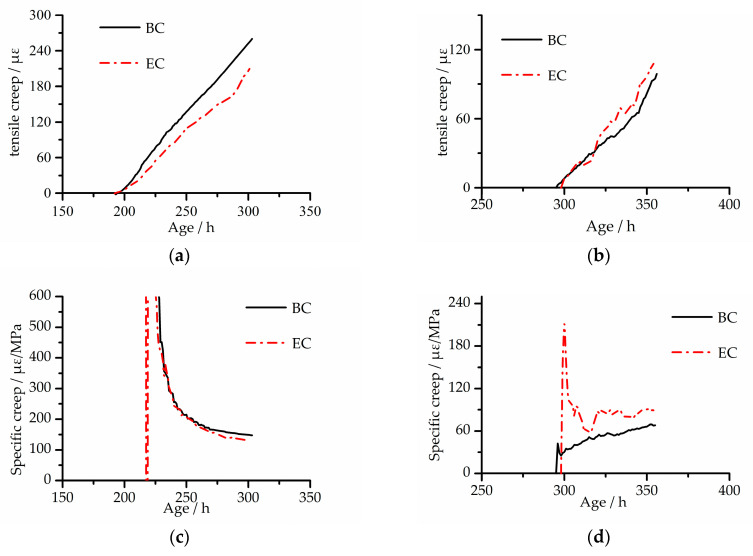
Tensile creep and specific creep of the two concretes under two modes: (**a**) tensile creep under temperature matched curing (TMC) mode; (**b**) tensile creep under constant temperature curing (CTC) mode; (**c**) specific creep under TMC mode; (**d**) specific creep under CTC mode.

The method of evaluating creep strain was suggested by Kovler [19]. He proposed that all the restored deformations from the compensation cycles could be accumulated into a cumulative curve that was exactly the total elastic strain, since the restored deformations were elastic. Based on this consideration, creep strain was calculated by subtracting the cumulative curve from the free shrinkage. Figure 5 shows schematically the principle of determining creep of concrete at early ages using the TSTM test. The calculation results of tensile creep and specific creep of the two concretes under two modes are shown in Figure 6. Formulas (1)–(3) are the creep calculation principle based on TSTM.

(1) $$\Delta \varepsilon_{\mathrm{th}}+\Delta \varepsilon_{\mathrm{au}}+\Delta \varepsilon_{\mathrm{cr}}+\Delta \varepsilon_{\mathrm{ce}}=0,$$

(2) $$\varepsilon_{\mathrm{th}}\left(t\right)+\varepsilon_{\mathrm{au}}\left(t\right)+\varepsilon_{\mathrm{cr}}\left(t\right)+\varepsilon_{\mathrm{ce}}\left(t\right)=0,$$

(3) $$\varepsilon_{\mathrm{cr}}\left(t\right)=-\left[\varepsilon_{f}\left(t\right)+\varepsilon_{\mathrm{ce}}\left(t\right)\right],$$

where ∆*ε*_th_, ∆*ε*_au_, ∆*ε*_cr_ and ∆*ε*_ce_ are thermal strain, autogenous strain, creep strain and elastic strain for each compensation cycle, respectively. *ε*_th_(*t*), *ε*_au_(*t*), *ε*_cr_(*t*) and *ε*_ce_(*t*) are the evolution of thermal strain, autogenous strain, creep strain and cumulative elastic strain, respectively, namely the accumulation of ∆*ε*_th_, ∆*ε*_au_, ∆*ε*_cr_ and ∆*ε*_ce_ for every compensation cycle. *ε*_f_(*t*) is free strain and equal to the sum of *ε*_th_(*t*) and *ε*_au_(*t*) as the restrained specimen and free specimen experienced the same curing temperature history.

As shown in Figure 6a,b, the EC and BC creep strain development rules are basically the same under both modes, and creep gradually increases with the growth of the loading time. The EC creep strain development curve is more tortuous than that of BC, which is especially obvious under CTC mode. The reason for the phenomenon was the adding of the MgO expansion agent. The MgO expansion agent still had an expansion effect when the specimens were in the cooling stage, which continuously reduced shrinkage.

**Figure 7:**
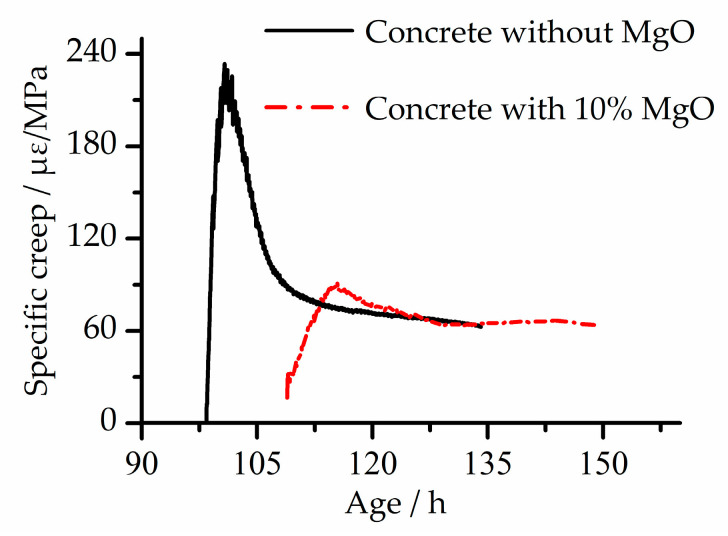
Specific creep of the two concretes under adiabatic mode adapted from [24].

As shown in Figure 6c,d, the specific creep of the two concretes is close under TMC mode; under CTC mode, the specific creep of EC is significantly larger than that of BC, and the final value is 31% larger than that of BC. During the cooling stage, because the specimens under the two modes did not fracture when the testing equipment reached the cooling limit, the specimens were pulled off manually by the motor. Therefore, the specific creep of the two concretes, as shown in Figure 6c,d, was not completely developed. For example, in Figure 6d, the specific creep of EC is stabilized, and the specific creep of BC tends to rise. Assuming that the temperature continues to drop, when the temperature reaches −15 °C, the stable value of specific creep eventually becomes the same. In order to verify this assumption, experimental data in the literature [24] were selected, and the specific creep of two kinds of concrete (concrete without the MgO expansion agent and concrete with the 10% MgO expansion agent) under adiabatic mode was obtained by calculation, as shown in Figure 7. In general, the MgO expansion agent had little effect on the specific creep of concrete.

In summary, the autogenous shrinkage of concrete mixed with MgO was compensated in the temperature stress test, which delayed the appearance of tensile stress and increased the cracking stress (Section 3.2). Correspondingly, the MgO concrete under standard curing had higher tensile strength and ultimate tension at an early age (Section 3.1). Regarding creep, the MgO expansion agent had little effect on the creep and specific creep. In our overall analysis, the early age cracking resistance of the concrete mixed with MgO was better than the benchmark concrete.

## 4. Improved Tensile Creep Models

### 4.1. The Kelvin Model Based on Degree of Hydration

**Figure 8:**
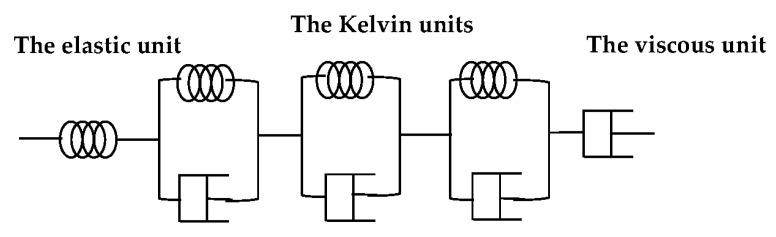
Kelvin creep model adapted from [17].

Combined with the measured data of the TST, the theoretical formula of the Kelvin model [17] under constant force was improved by the differential method with the variable stress formula. As shown in Figure 8, in the Kelvin creep model [17], the elastic unit represents the elastic deformation of concrete, the three Kelvin units represent the viscoelastic deformation of concrete and the viscous unit represents the viscous deformation of concrete. Among them, the degree of hydration determines the stiffness coefficient of the elastic unit, the stiffness coefficient and the viscosity coefficient of the Kelvin units and the viscosity coefficient of the viscous unit.

The constitutive equation of units is as follows:

1. The elastic unit:
  (4) $$\left\{ \begin{array}{l} \dot{\sigma }=E\left(\xi_{i}\right){\dot{\varepsilon }}_{e}\\ E\left(\xi \right)=E_{\infty }{\left[{\langle \frac{\xi \left(t\right)-\xi_{0}}{\xi_{\infty }-\xi_{0}}\rangle }_{+}\right]}^{0.62} \end{array}\right.,$$
  where *E*(*ξ*) is the modulus of elasticity based on the degree of hydration; *ε*_e_ is the strain of the elastic unit; *E*_∞_ represents the final modulus of elasticity; *ξ*(*t*) is the degree of hydration of the concrete at time *t*; *ξ*_0_ is the permeability threshold, generally taken as 0.1 [25], and we selected the degree of hydration corresponding to the initial setting time in this paper; *ξ*_∞_ represents the final degree of hydration; and <·>_+_ represents the absolute value.
2. The Kelvin units:
  (5) $$\left\{ \begin{array}{l} \dot{\sigma }={\dot{\sigma }}_{ki}+{\dot{\sigma }}_{\eta i}\\ {\dot{\sigma }}_{ki}=k_{i}\left(\xi \right){\dot{\varepsilon }}_{ki},\;i=1,2,3\\ \sigma_{\eta i}=\eta_{i}\left(\xi \right){\dot{\varepsilon }}_{ki} \end{array}\right.,$$
  where *σ* is the stress; *σ*_k*i*_ and *σ*_*ηi*_ are the stress of the elastic unit and viscous unit of the *i* Kelvin unit; *ε*_k*i*_ is the strain of the *i* Kelvin unit; and *k*_*i*_(*ξ*) and *η*_*i*_(*ξ*) are the function of the stiffness coefficient and viscosity coefficient of the *i* Kelvin unit with the development of hydration, and the value can be taken as follows [16]:
  (6) $$\left\{ \begin{array}{l} k_{i}(\xi )=k_{\infty }\frac{0.473}{2.081-1.608\bar{\xi }}{\bar{\xi }}^{0.62}\\ \eta_{i}(\xi )=k_{i}(\xi )\tau_{i}\\ \bar{\xi }=\frac{\xi (t)-\xi_{0}}{\xi_{\infty }-\xi_{0}} \end{array}\right.,$$
  where *τ*_i_ is the delay time of the *i* Kelvin unit and should meet a certain relationship [26]. In this paper, *τ*_1_ = 0.1 d, *τ*_2_ = 1 d and *τ*_3_ = 10 d.
3. The viscous unit:
  (7) $$\left\{ \begin{array}{l} \sigma =\eta_{a}\left(t\right){\dot{\varepsilon }}_{a}/\alpha \\ \eta_{a}\left(t\right)=k_{a}t \end{array}\right.,$$
  where *η*_a_(*t*) is the viscosity coefficient of the viscous unit, *ε*_a_ is the strain of the viscous unit, *α* is the coefficient of microcrack influence and *k*_a_ is a constant.

Combining with the slab concrete strain development of the TST, we show the discretized strain increment in Formula (8):

(8) $$\Delta \varepsilon^{n+1}={\Delta \varepsilon_{e}}^{n+1}+\sum_{i=1}^{3}{\Delta \varepsilon_{ki}}^{n+1}+{\Delta \varepsilon_{a}}^{n+1}.$$

The elastic strain increment obtained from Equation (4) is as follows:

(9) $$\Delta \varepsilon_{e}^{n+1}=\frac{\sigma^{n+1}-\sigma^{n}}{[E(\xi^{n+1})+E(\xi^{n})]/2},$$

The strain increment of the Kelvin units is obtained from Equations (5) and (6), as follows, where $\Delta t^{n+1}=t^{n+1}-t^{n}$.

(10) $$\Delta \varepsilon_{ki}^{n+1}=\frac{\Delta \sigma^{n+1}}{k_{i}^{av}(\frac{2\tau_{i}}{\Delta t^{n+1}}+\omega_{i})}+\frac{\tau_{i}{\dot{\varepsilon }}_{ki}^{n}}{\frac{\tau_{i}}{\Delta t^{n+1}}+0.5\omega_{i}},$$

where $k_{i}^{av}(\xi )=k_{i}^{av}\approx \frac{k_{i}(\xi^{n})+k_{i}(\xi^{n+1})}{2}$; ${\dot{\varepsilon }}_{ki}^{n}=2\frac{\Delta \varepsilon_{ki}^{n}}{\Delta t^{n}}-{\dot{\varepsilon }}_{ki}^{n-1}$ when the loading moment satisfies ${\dot{\varepsilon }}_{ki}^{0}=\frac{\sigma^{0}}{\eta_{i}}$, *i* = 1, 2, 3; $\omega_{i}=1+\frac{{\dot{k}}_{i}^{av}}{k_{i}^{av}}\tau_{i}$, where ${\dot{k}}_{i}^{av}(\xi )\approx \frac{k_{i}(\xi^{n+1})+k_{i}(\xi^{n})}{\Delta t^{n+1}}$.

From Equation (7), the strain increment of the viscous unit is as follows:

(11) $$\Delta \varepsilon_{a}^{n+1}=\alpha \frac{\sigma^{n+1}+\sigma^{n}}{2k_{a}}\ln\frac{t^{n+1}}{t^{n}},$$

For the improved Kelvin model, it is necessary to know the development history of stress and the degree of hydration and the calculation parameters of the model. We then obtain the tensile creep development of the slab concrete under restraint by iterative calculation of Formula (8).

### 4.2. Improved Kelvin Model Based on MPS Theory

Creep models are generally established under the constant temperature history. However, for the TST, because of the variable temperature with age, the accuracy of the prediction was affected.

According to the relationship between temperature and creep mentioned in Bazant’s microprestress-solidification (MPS) theory [13], we improved the Kelvin model [17]. In the theory, variable temperature affects creep by changing the microprestress in the micropores, and it mainly acts on the viscous unit of the Kelvin model. Therefore, the viscous unit is adjusted as follows.

(12) $$\left\{ \begin{array}{l} {\dot{\varepsilon }}_{a}=\frac{\sigma }{\eta \left(S\right)}\\ \frac{\dot{S}(t)}{C_{s}}+\frac{S(t)}{\eta \left(S\right)}=\frac{\dot{s}(t)}{C_{s}} \end{array}\right.,$$

where *ε*_a_ is the strain of the viscous unit; *η*(*S*) is the viscosity coefficient of the viscous unit; and 1/*η*(*S*) = *cbS*^*b*−1^, with *c* as the regression coefficient and *b* as 2 [27]; S(t) is microprestress; *Cs* is spring of stiffness; and $\dot{s}\left(t\right)$ indicates the variation of microprestress caused by the variable temperature and humidity.

Since the distances of water diffusion between the adjacent micropores and capillary pore are very short, the adsorbed water can establish thermodynamic equilibrium with the capillary water fast. Thus, it is assumed that all water phases locally are in thermodynamic equilibrium. This assumption follows the principle that Kelvin’s capillary equation is equal to the chemical potential in thermodynamics [13]. That is, the following equation is satisfied:

(13) $$s(t)=s_{1}-\frac{C_{1}RT}{M}(t)\ln\{h(t)\},$$

where *s*_1_ is a constant; *C*_1_ is a constant; *R* is the gas universal constant; M is the molecular weight of water; *T* is the absolute temperature; and *h*(*t*) is a function of humidity with time. The following formula is obtained by taking the derivative of Equation (13) with time.

(14) $$\dot{s}(t)=-k_{1}(\dot{T}\ln h+T\frac{\dot{h}}{h}),$$

where $k_{1}=C_{1}RT/M$.

By substituting Equation (14) into Equation (12), the microprestress formula for variable humidity and variable temperature is obtained as follows.

(15) $$\dot{S}+c_{0}S^{2}=-k_{1}(\dot{T}\ln h+T\frac{\dot{h}}{h}),$$

In this study, the concrete specimens were wrapped with plastic film, to prevent moisture exchange with the outside world. The humidity remains unchanged, so $\dot{h}$ is zero. A new viscous unit Equation (16) is obtained.

(16) $$\left\{ \begin{array}{l} {\dot{\varepsilon }}_{a}=\frac{\sigma }{\eta \left(S\right)}\\ \dot{S}+c_{0}S^{2}=k\dot{T} \end{array}\right.,$$

where $c_{0}=C_{s}cb$ and $k=\ln hk_{1}$.

From Equation (16), the strain increment of the viscous unit is as follows:

(17) $$\left\{ \begin{array}{l} \Delta \varepsilon_{a}^{n+1}=c(S_{n}+S_{n+1})(\sigma^{n+1}+\sigma^{n})\Delta t^{n+1}/2\\ S_{n+1}=\left[-c_{0}S_{n}^{2}+k\frac{T_{n+1}-T_{n}}{\Delta t^{n+1}}\right]/\Delta t^{n+1}+S_{n} \end{array}\right.,$$

### 4.3. The Degree of Hydration Curves

**Figure 9:**
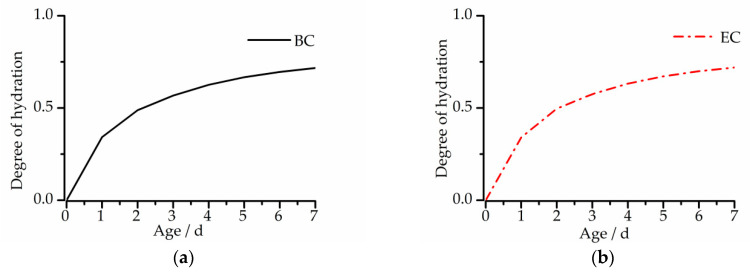
The degree of hydration curves of concrete: (**a**) BC and (**b**) EC.

For the calculation of the Kelvin model, it was necessary to gain the degree of hydration curves of cementitious concrete materials at an early age. The degree of hydration curves could be calculated from the hydration heat. Based on the China National Standard GB/T12959-2008 [28], we adopted the dissolution method to measure the early age hydration heat of cementitious concrete materials. The degree-of-hydration curves are shown in Figure 9.

### 4.4. Calculation Parameters of Two Models

Based on the least square method, the parameters of the tensile creep model were determined by analyzing the smallest difference between the model calculations and the actual measurements under the TMC mode. The calculation parameters of the Kelvin tensile creep model and improved model parameters based on MPS are shown in Table 5 and Table 6.

### 4.5. Verification of the Kelvin Model

**Figure 10:**
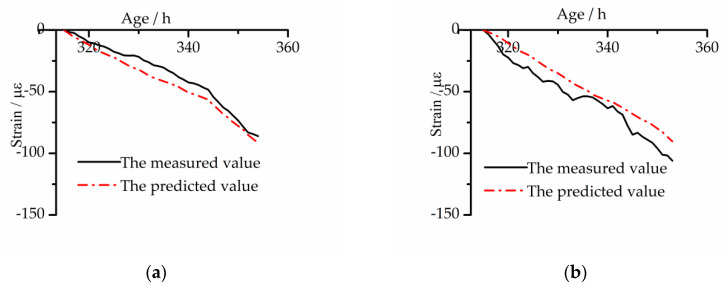
Comparison between the predicted free strain by the Kelvin tensile creep model and the measured one under CTC mode: (**a**) BC; (**b**) EC.

In order to check the accuracy of the model, the measured free strain of the early age concrete under the CTC mode was compared with the corresponding free strain prediction of the tensile creep model determined by the above parameters, as Figure 10 shows.

In order to reflect the accuracy of the model prediction more objectively, the maximum absolute value of the difference between the measured value and the predicted value was compared with the maximum measured value to obtain the maximum deviation ratio. As shown in Figure 9, under the CTC mode, the error range of the measured value and the predicted value of the BC is 1.6–10.8 με, and the maximum deviation ratio is 12.3%. The error range of the EC is 1.6–13.9 με, and the maximum deviation ratio is 13.3%.

### 4.6. Verification of Improved Kelvin Model under Variable Temperature

**Figure 11:**
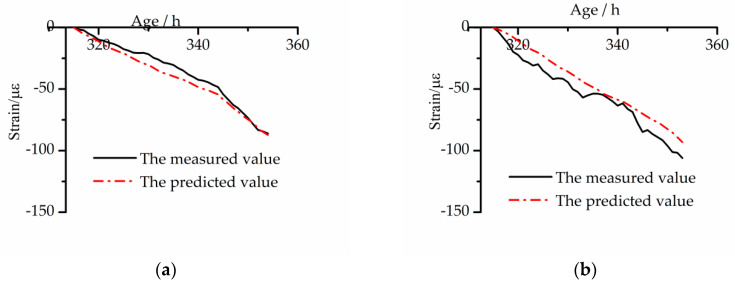
Comparison between the predicted free strain by the improved Kelvin tensile creep model and the measured one under CTC mode: (**a**) BC and (**b**) EC.

The accuracy of the improved model was tested with the method mentioned in Section 4.5. The results are shown in Figure 11.

As shown in Figure 11, the predicted values of the BC and EC have the same development trend as the test results, and the goodness of fit is good. The error range of the measured value and the predicted value of the BC is 1.4–7.8 με, and the maximum deviation ratio is 8.8%. The error range of the EC is 1.6–11.3 με, and the maximum deviation ratio is 9.3%.

Comparing the two models, we see the maximum deviation ratio of the BC is reduced from 12.3% to 8.8%, and the ratio of EC is reduced from 13.3% to 9.3%. This proves that the improved model based on MPS theory is more accurate in the prediction of tensile creep.

## 5. Conclusions

- In this study, the used expansive agent reduced shrinkage of slab concrete, delayed the appearance of the tensile stress and increased the cracking stress simultaneously, thus resulting in being beneficial to cracking resistance. The MgO expansion has little effect on creep and specific creep of concrete. In summary, the concrete mixed with MgO had better early age cracking resistance than the benchmark concrete.
- Two tensile creep models were proposed to predict the tensile creep development of slab concrete at early age accurately.
- Compared with the Kelvin creep model, the improved tensile creep model considering temperature variation has an obvious improvement in prediction accuracy. It preferably predicts the development of the early age tensile creep of slab concrete under variable temperature.

## Figures and Tables

**Table 1:** Mix proportions of benchmark concrete (BC) and concrete mixed with the expansion agent (EC).

Specimen	Unit Content (kg/m^3^)								
	Water	Cement	Fly Ash	MgO	Sand	Gravel (mm)		Water-Reducing Agent	Air-Entraining Agent
						**5–20**	**20–40**		
BC	120	188	63	0	768	541	661	1.506	0.0100
EC	120	188	63	12.5	763	537	657	1.750	0.0125

**Table 2:** Chemical composition of cementitious materials (%).

	CaO	Al_2_O_3_	MgO	SiO_2_	Fe_2_O_3_	SO_3_	LOI
Cement	58.62	5.23	2.05	23.21	3.70	2.78	2.44
Fly ash	5.25	28.18	0.79	46.65	5.43	0.71	3.29

**Table 3:** Performance indicators of the M-type MgO expansion agent.

	Absorption (%)	MgO Content (%)	LOI (%)	Reaction Time (s)	Setting Time (min)		Compressive Strength (MPa)	
					Initial	Final	7 Days	28 Days
M-type MgO	≤0.3	≥80.0	≤4.0	≥100 and <200	≥45	≤600	≥22.5	≥42.5

**Table 4:** Properties of fresh concrete.

Concrete	Setting Time (h)		Slump (mm)	Air Content (%)
	Initial	Final		
BC	10.3	13.4	80	4.3
EC	10.9	14.4	70	3.7

**Table 5:** Calculation parameters of the Kelvin tensile creep model.

Calculation Parameters	BC	EC
$k_{1\infty }/\mathrm{GPa}$	3	6
$k_{2\infty }/\mathrm{GPa}$	200	200
$k_{3\infty }/\mathrm{GPa}$	300	300
α	3	3
$K_{\alpha }/\mathrm{GPa}$	400	400

**Table 6:** Calculation parameters of improved Kelvin tensile creep model.

Calculation Parameters	BC	EC
$k_{1\infty }/\mathrm{GPa}$	3.5	6
$k_{2\infty }/\mathrm{GPa}$	40	50
$k_{3\infty }/\mathrm{GPa}$	300	300
k/MPa/K	3	3
$c/{\mathrm{MPa}}^{-2}d^{-1}$	7 × 10^−4^	7 × 10^−4^
$c_{0}/{\mathrm{MPa}}^{-1}d^{-1}$	1 × 10^−5^	8 × 10^−6^
